# A new functional classification system (FGA/B) with prognostic value for glioma patients

**DOI:** 10.1038/srep12373

**Published:** 2015-07-21

**Authors:** Katharina Friedlein, Yavor Bozhkov, Nirjhar Hore, Andreas Merkel, Björn Sommer, Sebastian Brandner, Michael Buchfelder, Nicolai E. Savaskan, Ilker Y. Eyüpoglu

**Affiliations:** 1 Department of Neurosurgery, Medical Faculty of the Friedrich Alexander University of Erlangen-Nürnberg (FAU)

## Abstract

Despite advances in multimodal treatments, malignant gliomas remain characterized by a short survival time. Surgical treatment is accepted to be the first line of therapy, with recent studies revealing that maximal possible tumor reduction exerts significant impact on patient outcome. Consideration of tumor localization in relation to functionally eloquent brain areas has been gaining increasing importance. Despite existing assessment methods, the availability of a simple but reliable preoperative grading based on functional data would therefore prove to be indispensable for the prediction of postoperative outcome and hence for overall survival in glioma patients. We performed a clinical investigation comprising 322 patients with gliomas and developed a novel classification system of preoperative tumor status, which considers tumor operability based on two graduations (Friedlein Grading - FG): FGA with lesions at safe distance to eloquent regions which can be completely resected, and FGB referring to tumors which can only be partially resected or biopsied. Investigation of outcome revealed that FGA were characterized by a significantly longer overall survival time compared to FGB. We offer the opportunity to classify brain tumors in a dependable and reproducible manner. The FGA/B grading method provides high prognostic value with respect to overall survival time in relation to the extent of location-dependent tumor resection.

Gliomas are primary CNS tumors accounting for almost 80% of all diagnosed tumors of the brain originating from brain parenchyma, with malignant gliomas constituting the most common brain tumors in adults^1^,^2^. Amongst them, glioblastoma (GBM, WHO°IV) carries the worst prognosis with patients succumbing after a median survival time of 14 months^3^. Although a cure remains elusive despite implementation of all currently available treatment options including radical surgical resection of the tumor mass followed by adjuvant radio-chemotherapy, this strategy serves to secure a histological diagnosis and improves response rates for radio-chemotherapy^4^,^5^. Maximizing cytoreduction by tumor resection through novel neurosurgical techniques still represents the first line therapy for glioma patients, with current trends focusing on the development of increasingly tailored treatment options with integration of molecular strategies in an attempt to at least maximize patient survival time and improve quality of life^6^,^7^,^8^.

A complete tumor resection has usually been understood to imply removal of the pre-operatively defined contrast-enhancing tumor portions. Whether such radical tumor resection exerts an influence on overall survival time was controversially discussed for a long time with some studies indicating no influence and others showing a positive correlation^9^,^10^,^11^,^12^,^13^,^14^. An important role behind this discrepancy can be traced back to patient distribution patterns based on an inherently heterogeneous tumor localization, which exerts a dramatic influence on the outcome of surgery with direct consequences for postoperative clinical course^8^,^15^. Thus, pre-operative tumor localization and subsequent operability is an important stratification parameter for proper clinical trials, something further complicated by considerable differences in individual surgical skill and center-specific state-of-the-art equipment^16^,^17^,^18^,^19^,^20^.

It is therefore clear that an easily accessible and dependable classification system is required meeting the needs for a pre- and postoperative tumor stratification accurately reflecting feasibility of resection. We describe a bipartite functional grading system (Friedlein grading A/B: FGA/B) based on functional topography aimed at creating a high degree of compliance for neuro-oncologists.

## Results

### Establishment of FGA/B as a novel prognostic classification system

**Figure 1:**
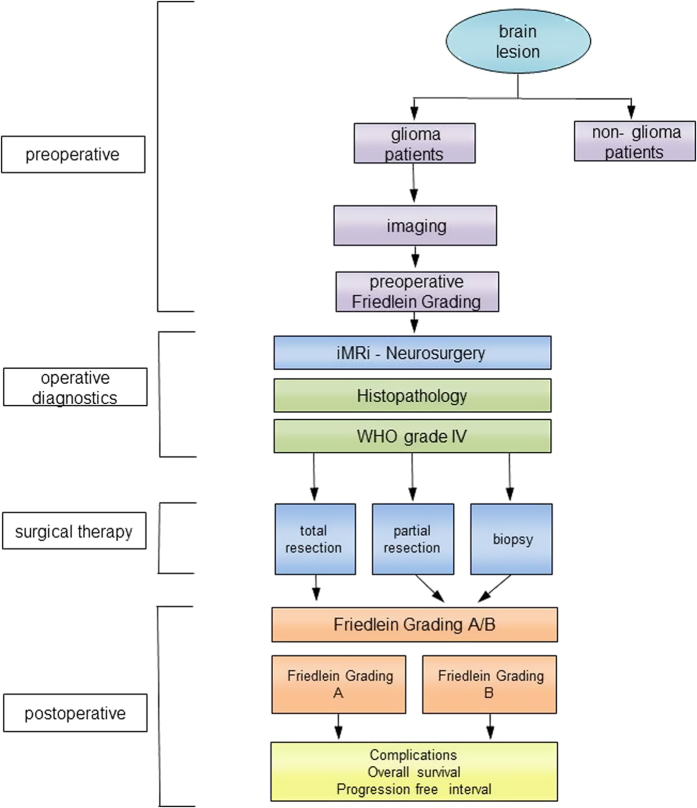
Clinical algorithm of the study. Only patients with suspicion of a high grade glioma based on initial screening of the presenting MRI scans were included in the study. These patients then underwent a preoperative functional imaging study with subsequent distribution into Friedlein grading system **A** (functionally silent area of the brain or adjacent to functionally eloquent areas of the brain) and **B** (in functionally eloquent areas of the brain). Following surgery, only patients with glioblastoma underwent further analysis with classification as Friedlein grading **A** (amenable to gross total resection) and Friedlein grading **B** (amenable to biopsy or at most subtotal resection). Following this, parameters like progression-free interval, overall survival time and postoperative complications were separately investigated.

Tumor localization and hence surgical accessibility are critical to successfully maximizing the extent of tumor resection. Therefore, we compared a novel classification system with an established one in order to develop a neuro-oncological classification tool with prognostic value. According to this algorithm we graded glioma patients on the basis of a four part procedure consisting of a pre-operative, histological, surgical and a postoperative step (Fig. 1). All patients with a brain lesion were divided into glioma and non-glioma cases depending on evaluation of initial presenting MRI scan with only suspected glioma patients undergoing subsequent functional MRI scans. According to these criteria we distributed patients into FGA/B: patients with FGA were characterized by suspected malignant glioma in a functionally silent area or near a functionally eloquent area (<8 mm). Patients with FGB were characterized by suspected malignant glioma in functionally eloquent or non-accessible areas. Only patients with histo-pathologically confirmed glioblastoma underwent further evaluation. Surgery was classified according to extent of tumor debulking into biopsy, subtotal tumor resection and gross total resection (>98% of the contrast-agent enhancing areas). For the purpose of data analysis, we then divided these tumor patients into the two distinct groups FGA/B. This was followed by documentation of clinical status, the rate of complications, Karnofsky Performance Scale, progression free interval and overall survival time (Fig. 1).

**Figure 2:**
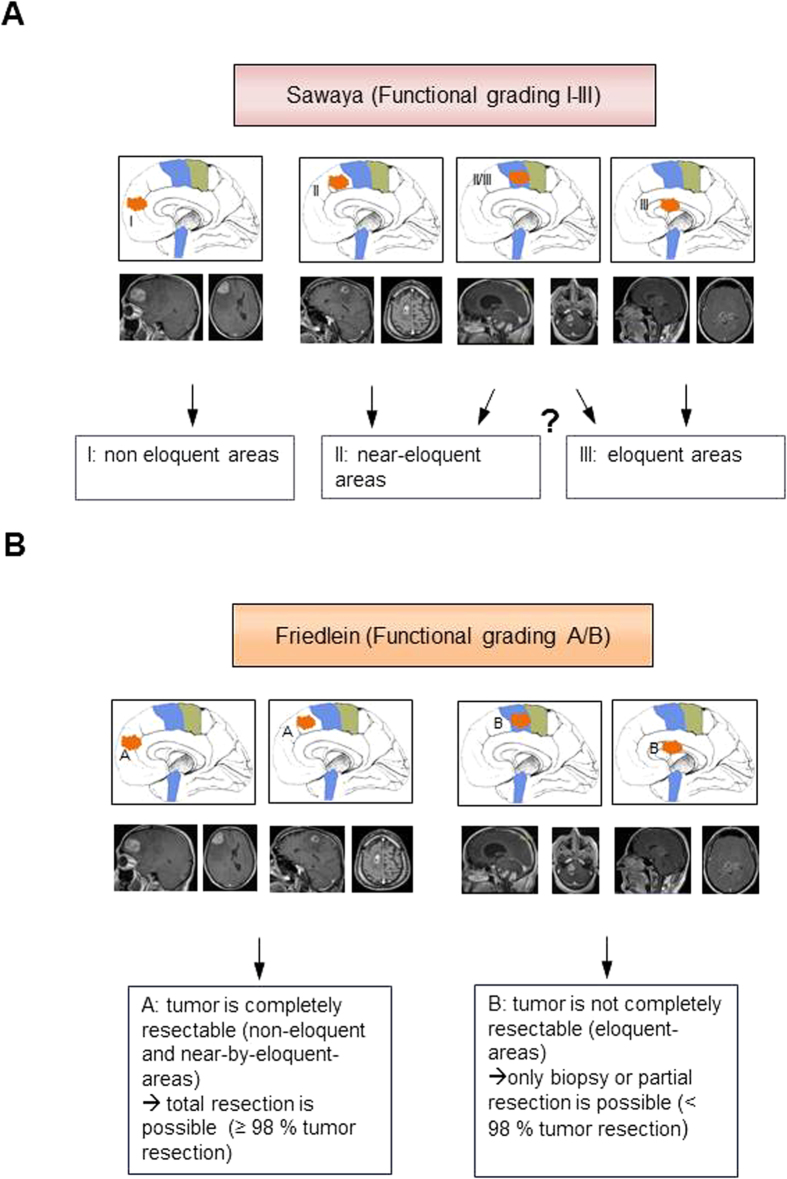
Tumor classifications according to the topography. (**A**) Description and sample MRI scans of Sawaya grading I – III. Sawaya I comprises tumors in functionally silent, Sawaya II near eloquent and Sawaya III in eloquent areas of the brain (**B**) Description and sample MRI scans of Friedlein grading A/B as a new prognostic classification. FGA comprises tumors both in functionally silent as well as near functionally eloquent areas of the brain, all of which can be subject to complete removal (>98% tumor resection), FGB comprises tumors in functionally eloquent areas of the brain and consequently subject to only biopsy or at best subtotal removal (<98% tumor resection). The drawings in the MRI sequences were performed by K.F.

For comparison, we then analyzed the same data with an existing classification system based on tumor localization in relation to functionally eloquent areas of the brain determined by topography alone, i.e. without confirmation through functional imaging studies^21^. Accordingly, patients were classified into the three functional groups I, II and III (Fig. 2A). Sawaya I comprises tumors located in functionally silent areas of the brain, whereas Sawaya II tumors are classified as tumors located adjacent to functionally eloquent areas of the brain. Sawaya III tumors are defined as tumors infiltrating functionally eloquent areas of the brain such as Broca’s, Wernicke’s area or the brainstem (Fig. 2A).

**Figure 3:**
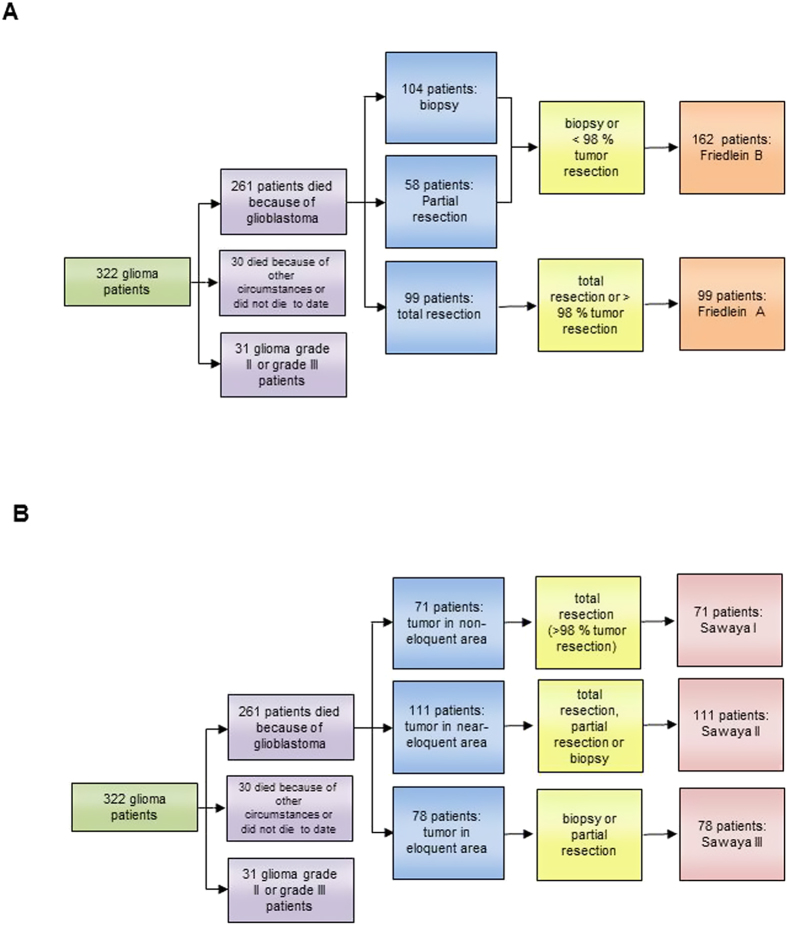
Comparative analysis of functional classification systems. The graph depicts the study design: 322 patients with suspected high grade glioma underwent functional MR imaging and all of them were histologically confirmed as glioma, of which only the 261 patients who succumbed due to glioblastoma progression underwent further analysis. The 30 patients who either died due to other circumstances like accident or surgical complications or were still alive at the end of the data acquisition period were excluded from further analysis. The remaining 31 patients suffering from low grade or anaplastic gliomas were also excluded from further analysis. Depending the 261 patients who succumbed to glioblastoma progression were then distributed according to extent of tumor resection into Friedlein grading (**A**) and Sawaya grading (**B**).

As a refinement, the Friedlein grading classifies tumors according to their resectability through precise analysis of their location in relation to functionally eloquent areas of the brain with verification of known topography through functional imaging (Fig. 2B). FGA therefore comprises all tumors subject to gross total resection (>98% tumor resection), whereas FGB comprises both tumors not amenable to gross total resection (<98% tumor resection) as well as those where only biopsy is possible. The pre- and postoperative patient cohort data has been correspondingly distributed into the subgroups according to FGA and FGB as well as Sawaya I-III and analyzed (Fig. 3). 99 patients were classified as FGA and 162 patients as FGB (Fig. 3A). In comparison 71 patients were classified as Sawaya I, 111 patients as Sawaya II, and 78 patients as Sawaya III (Fig. 3B).

Whereas a hallmark of the FGA/B system lies in a clear-cut distinction between the possibility of either complete or incomplete resection, corresponding to Sawaya I and III respectively, the presence of the distinctly heterogeneous group Sawaya II characterized by rather diverse extents of resection detracts significantly from the prognostic value of the Sawaya classification system.

### Pre- and postoperative patient characteristics and comorbidities

**Figure 4:**
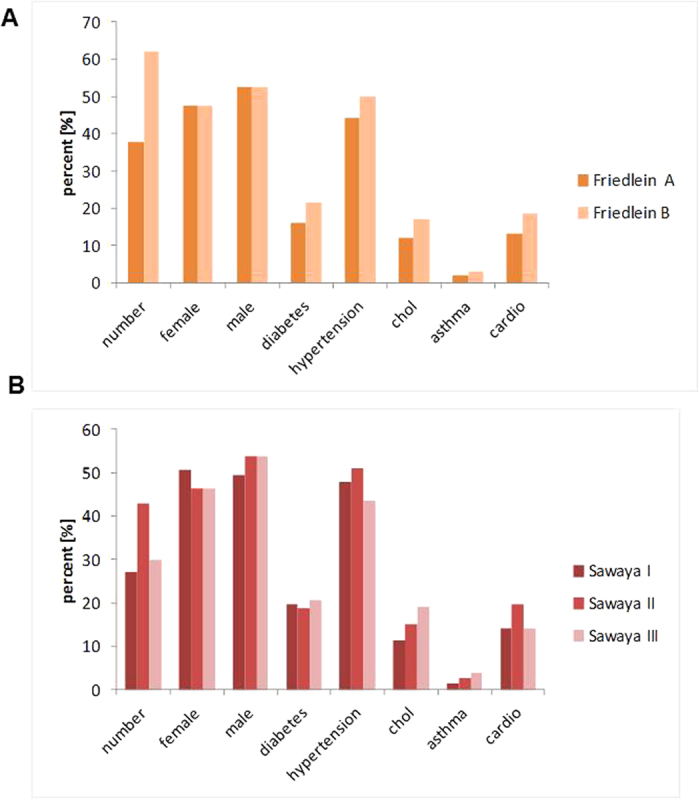
Analysis of patient cohort homogeneity. The two graphs depict the homogeneity of patients within FGA/B (**A**) and Sawaya I–III (**B**). In particular the distribution of comorbidities like diabetes, hypertension, hypercholesterolemia (chol), asthma and cardiopathies (cardio) were analyzed as well as patient numbers and gender distribution.

322 patients underwent surgery, and in all cases postoperative MRI scans were performed. 261 patients were histologically diagnosed as glioblastoma WHO grade IV, 31 of WHO Grade II-III and 30 died because of other circumstances, i.e. an accident or during anesthesia and surgery or were still surviving. Average patient age was 61.8 ± 13.9 years at the time of surgery. Median survival time was 352.5 days (average survival time 538.0 ± 785.1 days). 122 (46.7%) suffered from hypertension, 52 (19.9%) from diabetes, 42 (16.1%) from cardiopathies, 39 (14.9%) from hypercholesterolemia and 7 (2.7%) from bronchial asthma. 73 patients developed a recurrent tumor. A comparative analysis of the two classification systems revealed that both patient cohorts were characterized by a similar and normal distribution with respect to sex, age and comorbidities (Fig. 4). Homogeneity was also tested with respect to diabetes mellitus, hypertension, hypercholesterolemia, bronchial asthma, cardiopathies, BMI, radio-chemotherapy and patient age. Average age of Sawaya I was 61.1 ± 10.8, of Sawaya II 62.3 ± 11.5 and Sawaya III 61.9 ± 12.1 years, while the average age of the Friedlein groups was 60.9 ± 10.9 for FGA and 62.4 ± 12.8 for FGB years reflecting a homogenous patient distribution in both classification systems (Fig. 4).

### Survival analysis according to Friedlein and Sawaya

**Figure 5:**
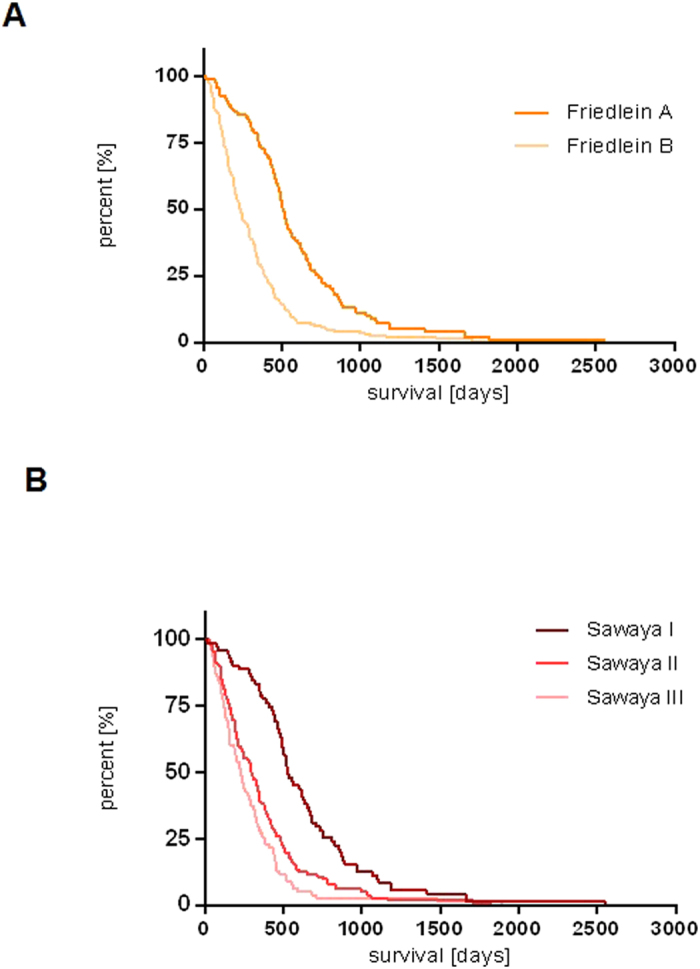
Analysis of overall survival time in both classification systems. Overall survival time of FGA/B (A) and Sawaya I - III (B) were depicted as Kaplan-Meier curves. FGA patients were characterized by a significantly prolonged overall survival time compared to FGB (Log-rank p-value < 0.0001). Sawaya I patients were characterized by a significantly prolonged overall survival time in comparison to Sawaya II and III patients (p-value < 0.0001 and <0.0001). In contrast Sawaya II patients were characterized by a lack of significant prolongation of overall survival time in comparison to Sawaya III patients (p-value 0.521).

**Figure 6:**
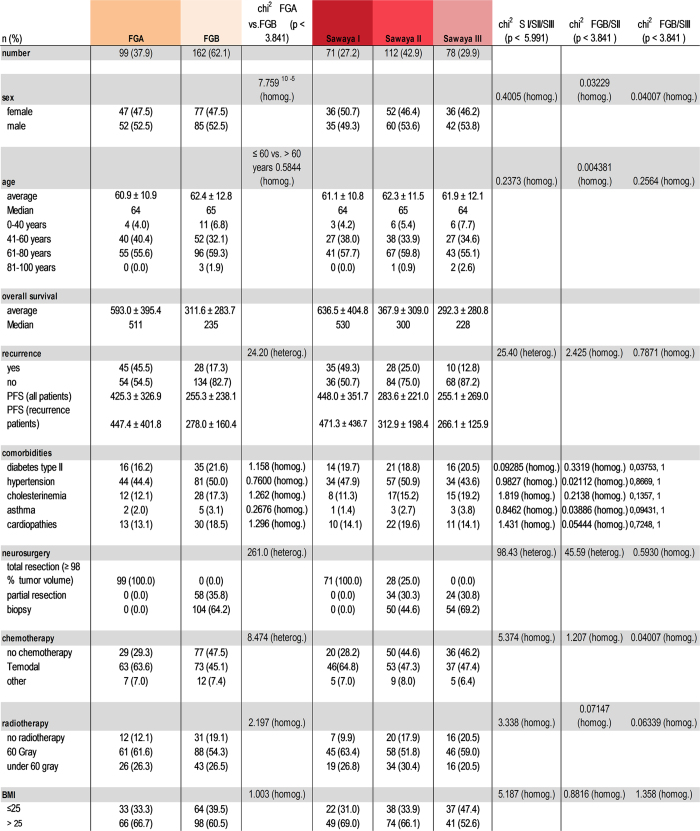
Statistical analysis of clinical parameters and distributions. Pre- and postoperative characteristics and comorbidities of patients included in this study.

The median survival time of FGA was 511 days (mean overall survival time 593.0 ± 395.4 days) and of FGB was 235 days (mean overall survival time 311.6 ± 283.7 days). In comparison, the corresponding median survival time of Sawaya I was 530 days (mean overall survival time 636.5 ± 404.8 days), of Sawaya II was 300 days (mean overall survival time 367.9 ± 309.0 days) and of Sawaya III was 228 days (mean overall survival time 292.3 ± 280.8 days). Survival analysis of Friedlein grading (Fig. 5A) and Sawaya grading (Fig. 5B) are presented in the form of Kaplan-Meier curves. Patients of FGA demonstrated a significant increase of overall survival compared to patients included in the FGB group (log rank p-value < 0.0001; HR 0.4592; 95% CI 0.3400 to 0.5551). In comparison patients of Sawaya I lived significantly longer than patients of Sawaya II and III (log rank p-value < 0.0001; HR 0.5063; 95% CI 0.3668 to 0.6572 and log rank p-value < 0.0001; HR 0.3793; 95% CI 0.2081 to 0.4210). In contrast, patients of Sawaya II were characterized by no significant prolongation in overall survival time compared to Sawaya III (log rank p-value 0.521; 95% CI 0.5486 to 0.9981). A comparison between FGA and Sawaya II patients showed a significant increase in overall survival time for FGA (log rank p-value: <0.0001; HR 0.5467; 95% CI 0.3934 to 0.6855). A comparison between FGB and Sawaya II showed no significant difference in overall survival time (log rank p-value 0.096; HR 1.223; 95% CI 0.9669 to 1.555). A summary of the pre- and postoperative characteristics and comorbidities of all included patients is shown in Fig. 6.

**Figure 7:**
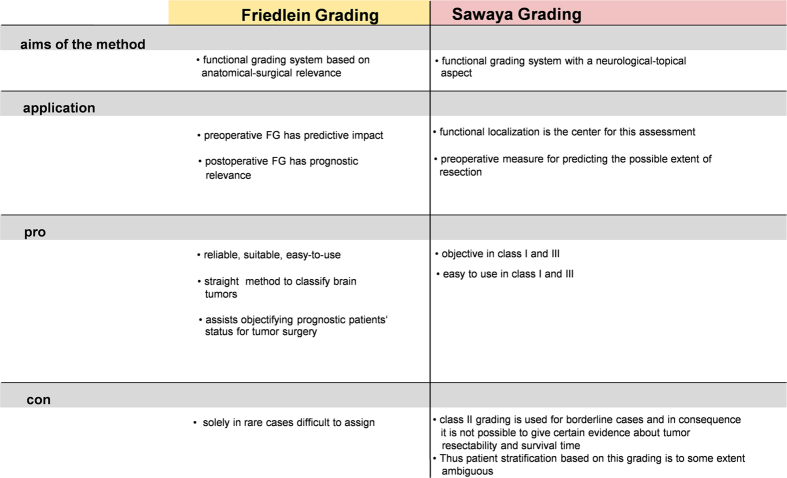
Pros and cons of the Friedlein and Sawaya grading system. Advantages and disadvantages of Friedlein and Sawaya grading classifications with special regard to the peculiarity of Sawaya II patients as well as the aims and application method of both classification systems.

**Figure 8:**
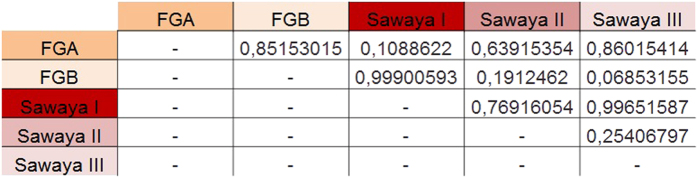
Analysis of effect sizes between FG and Sawaya groups. Effect sizes were calculated with Cohen’s d. A weak effect was defined as d < 0.2, a mean effect as d = 0.2 to 0.8 and a strong effect as d > 0.8.

**Figure 9:**
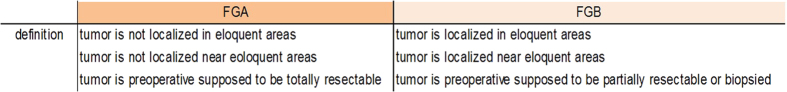
Definitions of Friedlein grading A/B. FGA/FGB classification on the basis of tumors localization and preoperative MRI.

Both classification systems were compared with analysis of respective pros and cons (Fig. 7). Further analysis of the effect sizes of Friedlein and Sawaya (Fig. 8) showed a strong effect between FGA and FGB (d = 0.85153015) and between Sawaya I and Sawaya III (d = 0.99651587), a mean effect between Sawaya I and Sawaya II (d = 0.76916054) and a weak effect between Sawaya II and Sawaya III (d = 0.25406797). These results confirm the assumption that FGA/B grading provides high prognostic value for overall survival time whereas Sawaya II remains ambiguous in this respect: allocation to FGA would indicate the possibility of gross total resection whereas allocation to FGB would indicate the possibility of either a biopsy or subtotal resection at best due to eloquent localization, implying in this case tumor residue visible in MRI scans (Fig. 9).

## Discussion

Due to modest improvements in neuro-oncological treatment, refinement of prognostic and predictive factors is essential for stratifications of patient cohorts with the aim to establish an individually customized and balanced therapy^5^,^22^,^23^. Such prognostic and predictive factors exercise decisive influence on the course of treatment of oncological patients and are therefore expected to improve the efficacy of tailored therapy regimes. Whereas to this respect numerous epidemiological parameters have already been defined, the number of factors known to have an impact on life expectancy in malignant gliomas is very limited. Amongst them, the most popular predictive factors are the MGMT methylation status and the presence of an IDH1 mutation^24^,^25^,^26^,^27^. These parameters are predictive for success of adjuvant, individually tailored therapies such as the response rate for temozolomide^28^,^29^.

Precise information about the molecular nature of the tumor obtained through surgery plays a key role in determining individualized post-surgical therapy options. It is also likely to play a significant future role in the stratification of patients. Amongst prognostic factors, age and general condition are considered to be the primary limiting factors^30^,^31^,^32^. Patients of young age and in good general condition as measured by the Karnofsky Performance Scale are correspondingly expected to be characterized by a longer survival time^33^. It ought to be mentioned here that both preoperative poor general condition as well as an iatrogenically induced deterioration equally contribute to a dramatic decrease in quality of life and overall survival time. These factors must therefore be taken into due consideration in the development of treatment options for neuro-oncological patients in order to be able to offer tailored solutions. The question remains to be addressed as to why such great variability in reported outcomes exists despite neurosurgical tumor resection uniformly setting the first line therapy in malignant gliomas.

Despite the extent of resection having been proven to impact patient response to adjuvant therapy and overall survival time^8^,^15^,^34^, several large clinical trials performed on GBM cohorts failed to take surgical outcome as defined by postoperative quality of life into consideration and did not systematically evaluate the relevance of residual tumor volume^30^,^35^. Other investigations have demonstrated that the inclusion of extent of resection and remaining tumor load as stratification parameters is characterized by a high prognostic value^7^,^36^,^37^.

The extent of resection depends on the accessibility of the tumor as defined by its relation to functional eloquent areas of the brain, which is why objective methods were developed to define this topography^8^,^38^,^39^. As in many other centers, these functional eloquent areas are identified according to preoperative functional MR imaging, in our department too. It is important to mention at this point that another powerful surgical technique called intraoperative functional mapping can be implemented in surgery in the vicinity of functionally eloquent brain areas^40^,^41^,^42^. This form of surgery is not universally in use—many centers prefer preoperative visualization of functional areas with surgery carried out on the basis of this functional neuronavigation without intraoperative stimulation^43^,^44^,^45^. This could in principle be considered a disadvantage of our study, as it orients towards the functional areas with iMRI but without awake surgery. It ought to be investigated in a separate study whether the combination of intraoperative MRI with integrated functional neuronavigation and intraoperative functional mapping could lead to a still further increase in the radicalness of resection. Nevertheless, it is necessary to develop simpler methods of preoperative planning to increase accessibility of this technique. One such attempt to develop a simple system was the three-grade Sawaya classification^21^. Although this classification system is valuable in surgical planning for tumors either in functionally silent areas of the brain (Sawaya I) or infiltrating functionally eloquent areas (Sawaya III), an inevitable degree of ambiguity surrounds tumors adjacent to functionally eloquent areas (Sawaya II) which is corroborated by the lack of significant difference in overall survival time between Sawaya II and III. This stems from the fact that the functionally eloquent areas of the brain were defined on the basis of prior anatomical knowledge without confirmation through functional imaging studies. On the other hand, the FGA/B grading system is characterized by a clear-cut system based on the verification of anatomically known functionally eloquent areas of the brain with the corresponding possibility of precise determination of resectability. The prognostic value of the FGA/B grading system further becomes apparent due to the possibility of individual observation of the clearly different clinical courses of the patient subgroups.

FGA/B is therefore an easily implementable and dependable classification system with high prognostic value satisfying the criteria for a preoperative stratification of GBM patients with high compliance for neuro-oncologists. Integration of the FGA/B grading system could therefore play an important role in the refinement of patient stratification in future clinical trials.

## Material & Methods

### Clinical data

The study was approved by the local ethics committee of the University of Erlangen-Nürnberg and complies with the current laws of the Federal Republic of Germany. All clinical investigations were conducted in accordance with the Declaration of Helsinki. The study consists of clinical evaluation as well as pre- and intraoperative MRI scans with subsequent data analysis. Prior informed consent was obtained in writing from each patient included in the study. Further details pertaining to the cohort consisting of 261 patients are elaborated in Fig. 6. All methods were carried out in accordance with the approved guidelines.

### Glioblastoma cohort

From a total of 322 patients 261 with histopathologically confirmed supra-tentorial GBM operated with intraoperative MRI from October 2000 to October 2011 were included in this study. Patient age at time of surgery ranged between 11 and 84 years. Patient cohort consisted of 124 female and 137 male patients. Postoperative survival time was calculated in days retrospectively obtained from the Erlangen Tumor Register database (Tumorzentrum Mittelfranken) and ranged from 9 to 1869 days following initial diagnosis. Symptoms taken into consideration included headache, personality and orientation disturbances, epileptic seizures, motor deficits, sensory deficits, speech disturbances, and visual field deficits. Comorbidities documented were arterial hypertension, hypercholesterolemia, diabetes mellitus, gastrointestinal diseases, mental diseases, cardiopathies, coagulopathies, bronchial asthma, thyroid dysfunction, osteoporosis and venous thrombosis.

### Tumor volumetry

Tumor segmentation and postoperative volumetric analysis were performed with the VectorVision planning software on an offline workstation. Data was transferred with the help of the PatXfer data-transfer software (BrainLAB). Tumor volume was calculated in cubic centimeters following manual tumor segmentation across all appropriate slices (T1 with contrast enhancement + T2). Tumor volumes ranged from 0.5 cm^3^ to 108.7 cm^3^.

### Friedlein grading A/B system

The distinction of Friedlein grading A/B is clear and easy to handle. A tumor is defined as FGA if the tumor is not localized within or near eloquent areas and it is preoperatively (on the basis of MRI) assumed that the tumor can be totally resected. If a tumor is localized near or within eloquent areas and preoperatively (on the basis of MRI) supposed to be partially resectable or can only be biopsied it is assigned to FGB (Fig. 9).

### Statistical analysis

Statistical significance was calculated with GraphPad Prism v6.05. The survival time curves were statistically analyzed with the Mantel-Cox (logrank) and the Gehan-Breslow-Wilcoxon Tests. The Mantel-Cox (logrank) test was used as a common hypothesis-test to compare the survival distributions of two or three groups. Hazard ratios (HRs) and their adjusted 95% confidence intervals (CIs) were calculated. Normal distribution and homogeneity were investigated with Chi-square-test (α = 0.05 (level of significance 95%)). The Chi-square-test was used as a statistical hypothesis test for independence and homogeneity in groups. P-values < 0.05 were considered to be statistically significant. BMI, localization of surgery, patient age, comorbidities (diabetes, asthma, hypercholesterolemia, coagulopathies and hypertension) and radiotherapy were independent factors. In contrast, surgical treatment and chemotherapy in FG groups were dependent factors. The effect size of the study was analyzed with Cohen’s d. A weak effect was given for d < 0.2, a mean effect for d = 0.2 to 0.8 and a strong effect for d > 0.8.

## Additional Information

**How to cite this article**: Friedlein, K. *et al.* A new functional classification system (FGA/B) with prognostic value for glioma patients. *Sci. Rep.* **5**, 12373; doi: 10.1038/srep12373 (2015).
